# Supplementary material for: Otolith microchemistry and diadromy in Patagonian river fishes
Source: PeerJ. 2019 Jan 3;7:e6149. doi: 10.7717/peerj.6149 (PMC6321752; doi:10.7717/peerj.6149)
Supplement: Supplemental Information 1 — The following table lists the native continental fishes of Chile, highlighting current knowledge about their preferred habitat and/or life history. Abbreviations in the Life history/habitat column correspond to: A, anadromous; C, catadromous; Am, amphidromous; P, potadromous; FW, obligate freshwater resident; E, euryhaline; SW, indicates either estuarine preference or pseudo complete ocean residency. [file peerj-07-6149-s001.docx]

**TABLE S1**

The following table lists the native continental fishes of Chile, highlighting current knowledge about their preferred habitat and/or life history. Abbreviations in the Life history/habitat column correspond to: A, anadromous; C, catadromous; Am, amphidromous; P, potadromous; FW, obligate freshwater resident; E, euryhaline; SW, indicates either estuarine preference or pseudo-complete ocean residency.

| FAMILY | GENUS | SPECIES | LIFE HISTORY/HABITAT | GEOGRAPHIC RANGE (Origin) | REFERENCES |
| --- | --- | --- | --- | --- | --- |
| Petromyzontidae | *Geotria* | *australis (Gray, 1851)* | A | Southern Hemisphere | (McDowall 1988; Neira et al. 1988) |
| Petromyzontidae | *Mordacia* | *lapicida (Gray, 1851)* | A | Chile | (McDowall 2002) |
| Characidae | *Cheirodon* | *pisciculus (Girard, 1855)* | FW | Chile | (Salas et al. 2012) |
| Characidae | *Cheirodon* | *australe (Eigenmann, 1927)* | FW | Chile | (Salas et al. 2012) |
| Characidae | *Cheirodon* | *galusdae (Eigenmann, 1928)* | FW | Chile | (Salas et al. 2012) |
| Characidae | *Cheirodon* | *kiliani (Campos, 1982)* | FW | Chile | (Salas et al. 2012) |
| Diplomystidae | *Diplomystes* | *chilensis (Molina, 1782)* | FW | Chile | (Munoz-Ramirez et al. 2010) |
| Diplomystidae | *Diplomystes* | *camposensis (Arratia, 1987)* | FW | Chile | (Munoz-Ramirez et al. 2010) |
| Diplomystidae | *Diplomystes* | *nahuelbutaensis (Arratia, 1987)* | FW | Chile | (Munoz-Ramirez et al. 2010) |
| Trichomycteridae | *Nematogenys* | *inermis (Guichenot, 1848)* | FW | Chile | (Habit and Victoriano 2005; Vargas et al. 2015) |
| Trichomycteridae | *Trichomycterus* | *areolatus (Valenciennes, 1846)* | FW | Chile | (Unmack et al. 2009) |
| Trichomycteridae | *Trichomycterus* | *chiltoni (Eigenmann, 1928)* | FW | Chile | (Habit and Victoriano 2005) |
| Trichomycteridae | *Trichomycterus* | *rivulatus (Valenciennes, 1846)* | FW | Chile, Peru, Bolivia | (Pardo et al. 2005; Habit et al. 2006) |
| Trichomycteridae | *Trichomycterus* | *chungaraensis (Arratia 1983)* | FW | Chile | (Arratia F. 1983; Vargas et al. 2015) |
| Trichomycteridae | *Trichomycterus* | *laucaensis (Arratia 1983)* | FW | Chile | (Arratia F. 1983; Vargas et al. 2015) |
| Trichomycteridae | *Hatcheria* | *macraei (Berg, 1985)* | FW | Chile, Argentina | (Unmack et al. 2012; Vargas et al. 2015) |
| Trichomycteridae | *Bullockia* | *maldonadoi (Eigenmann, 1920)* | FW | Chile | (Habit and Victoriano 2005) |
| Galaxiidae | *Galaxias* | *maculatus (Jenyns, 1842)* | C | Southern Hemisphere | (Górski et al. 2015; Vargas et al. 2015) |
| Galaxiidae | *Galaxias* | *globiceps (Eigenmann, 1928)* | FW | Chile | (Murillo & Ruiz 2002) |
| Galaxiidae | *Galaxias* | *platei (Steindachner, 1898)* | FW | Chile, Argentina | (Ruzzante et al. 2008; Vargas et al. 2015) |
| Galaxiidae | *Brachygalaxias* | *bullocki (Regan, 1908)* | FW | Chile | (Habit and Victoriano 2005; Correa-Araneda et al. 2014; Vargas et al. 2015) |
| Galaxiidae | *Brachygalaxias* | *gothei (Busse, 1983)* | FW | Chile | (Cuevas et al. 1999; Vargas et al. 2015) |
| Galaxiidae | *Aplochiton* | *zebra (Jenyns, 1842)* | FW | Chile, Argentina, Malvinas | (Vargas et al. 2015) |
| Galaxiidae | *Aplochiton* | *taeniatus (Jenyns, 1842)* | C | Chile, Argentina, Malvinas | (Vanhaecke et al. 2012; Alò et al. 2013; Vargas et al. 2015) |
| Galaxiidae | *Aplochiton* | *marinus (Eigenmann, 1928)* | C | Chile | (Alò et al. 2013; Vargas et al. 2015) |
| Atherinopsidae | *Basilichthys* | *microlepidotus (Jenyns, 1841)*(syn:*australis)* | FW | Chile | (Dyer 2000) |
| Atherinopsidae | *Basilichthys* | *semotilus (Cope, 1874)* | P | Chile, Peru | (Dyer 2000; Vargas et al. 2015) |
|  |  |  |  |  |  |
| Atherinopsidae | *Odontesthes* | *mauleanum (Steindachner, 1896)* | E | Chile | (Dyer 2000) |
| Atherinopsidae | *Odontesthes* | *itatanum (Steindachner, 1896)* | E | Chile | (Dyer 2000) |
| Atherinopsidae | *Odontesthes* | *brevianalis (Gunther, 1880)* | E | Argentina, Chile | (Dyer 2000; Ministerio del Medio Ambiente 2013) |
| Atherinopsidae | *Odontesthes* | *hatchery* (Eigenmann, 1909) | E | Argentina, Chile | (Tsuzuki et al. 2008; Ministerio del Medio Ambiente 2013) |
| Atherinopsidae | *Odontesthes* | *molinae (Fowler, 1940) or (Fischer, 1962)* | No information (Possible synonym with other Odontesthes) | Chile | (Campos et al. 1998; Vargas et al. 2015) |
| Atherinopsidae | *Odontesthes* | *regia* (Humboldt, 1821) | SW (Juveniles in estuarine) | Peru, Chile, Argentina, Malvinas | (Ministerio del Medio Ambiente 2013) |
| Atherinopsidae | *Odontesthes* | *nigricans* (Richardson, 1848) | SW (Juveniles in estuarine) | Chile, Argentina, Malvinas | (Dyer 2000) |
| Atherinopsidae | *Odontesthes* | *wiebrichi* (Eigenmann, 1928) | E (Possible hybrid regia/brevianalis) | Chile | (Dyer 2000) |
| Cyprinodontidae | *Orestias* | *agassii (Valenciennes, 1846)* | FW | Peru, Chile | (Diario Oficial de la Republica de Chile 2008; Vargas et al. 2015) |
| Cyprinodontidae | *Orestias* | *chungarensis (Arratia, 1982)* | FW | Chile | (Diario Oficial de la Republica de Chile 2008; Vargas et al. 2015) |
| Cyprinodontidae | *Orestias* | *parinacotensis (Arratia, 1982)* | FW | Chile | (Diario Oficial de la Republica de Chile 2008; Ministerio del Medio Ambiente 2013; Vargas et al. 2015) |
| Cyprinodontidae | *Orestias* | *laucaensis (Arratia, 1982)* | FW | Chile | (Diario Oficial de la Republica de Chile 2008; Ministerio del Medio Ambiente 2013) |
| Cyprinodontidae | *Orestias* | *ascotanensis (Parenti, 1984)* | FW | Chile | (Pardo et al. 2005; Vila et al. 2007; Diario Oficial de la Republica de Chile 2008; Ministerio del Medio Ambiente 2013) |
| Cyprinodontidae | *Orestias* | *gloriae (Vila et al, 2011)* | FW (Saltpans) | Chile | (Vila et al. 2011) |
| Cyprinodontidae | *Orestias* | *piacotensis (Vila, 2006)* | FW | Chile | (Vila 2006; Ministerio del Medio Ambiente 2013) |
| Mugilidae | *Mugil* | *cephalus (Linnaeus, 1758)* | E/50km upstream FW | Worldwide coastal areas | (Habit and Victoriano 2005; Diario Oficial de la Republica de Chile 2008; Ministerio del Medio Ambiente 2013; Vargas et al. 2015) |
| Percichthyidae | *Percichthys* | *melanops (Girard, 1855)* | FW | Chile | (Habit and Victoriano 2005; Diario Oficial de la Republica de Chile 2008; Ministerio del Medio Ambiente 2013; Vargas et al. 2015) |
| Percichthyidae | *Percichthys* | *trucha (Regan, 1905)* | FW | Argentina, Chile | (Ruzzante et al. 2006; Diario Oficial de la Republica de Chile 2008; Vargas et al. 2015) |
| Perciliidae | *Percilia* | *gillissi (Girard, 1855)* | FW | Chile | (Habit and Victoriano 2005; Ministerio del Medio Ambiente 2013; Vargas et al. 2015) |
| Perciliidae | *Percilia* | *irwini (Eigenmann, 1928)* | FW | Chile | (Habit and Victoriano 2005; Diario Oficial de la Republica de Chile 2008; Ministerio del Medio Ambiente 2013) |

**References**

Alò D, Correa C, Arias C, Cárdenas L (2013) Diversity of Aplochiton Fishes (Galaxiidea) and the Taxonomic Resurrection of *A. marinus*. PLoS One 8:e71577. doi: 10.1371/journal.pone.0071577

Arratia F. G (1983) *Trichomycterus chungaraensis* n. sp. and *Trichomycterus laucaensis* n. sp. (Pisces, Siluriformes, Trichomycteridae) from the high Andean range. Stud Neotrop Fauna Environ 18:65–87. doi: 10.1080/01650528309360621

Campos HC, Dazarola G, Dyer BS, et al (1998) Categorias de conservacion de peces nativos de aguas continentales de Chile. Bol del Mus Nac Hist Nat 47:101–122.

Correa-Araneda F, De Los Ríos P, Habit E (2014) Presence of the red jollytail, *Brachygalaxias bullocki* (Regan, 1908) (Galaxiformes: Galaxiidae), in freshwater forested wetlands from Chile. Rev Chil Hist Nat 87:1–4. doi: 10.1186/s40693-014-0020-4

Cuevas C, Campos H, Busse K (1999) Cytotaxonomic studies on Chilean Galaxiid fishes. The kariotypes, C-bands, Ag-NORs and Hybrids of *Brachygalaxias gothei* and *B. bullocki* (Osteichthyes: Galaxiidae). Cytologia (Tokyo) 64:379–385.

Diario Oficial de la Republica de Chile (2008) Tercer Proceso de Clasificacion de Especies segun su Estado de Conservacion.

Dyer BS (2000) Systematic review and biogeography of the freshwater fishes of Chile. Estud Ocean 19:77–98.

Górski K, Habit EM, Pingram MA, Manosalva AJ (2015) Variation of the use of marine resources by *Galaxias maculatus* in large Chilean rivers. Hydrobiologia. doi: 10.1007/s10750-015-2542-4

Habit E, Dyer BS, Vila I (2006) Current state of knowledge of freshwater fishes of Chile. Gayana 70:100–113.

Habit EM, Victoriano P (2005) Peces de agua dulce de la Cordillera de la Costa. In: Biodiversidad y Ecología de la Cordillera de la Costa de Chile. pp 374–389

McDowall RM (1988) Diadromy in Fishes. Timber Press, Portland, Oregon

McDowall RM (2002) Accumulating evidence for a dispersal biogeography of southern cool temperate freshwater fishes. J Biogeogr 29:207–219. doi: 10.1046/j.1365-2699.2002.00670.x

Ministerio del Medio Ambiente (2013) Especies: Clasificacion segun estado de conservacion. http://www.mma.gob.cl/clasificacionespecies/doc/UNIFICADA_de_Especie_Estado_Conservacion_Nov2014_publico.xls.

Munoz-Ramirez C, Jara A, Beltran-Concha M, et al (2010) Distribucion de la familia Diplomystidae (Pisces: Siluriformes) en Chile: Nuevos Registros. Bol Biodivers Chile 4:6–17.

Murillo & Ruiz (2002) El Puye *Galaxias Globiceps*. Gayana 66:191–197. doi: 10.13140/RG.2.1.5180.0481

Neira FJ, Bradley JS, Potter IC, Hilliard RW (1988) Morphological variation among widely dispersed larval populations of anadromous southern hemisphere lampreys (Geotriidae and Mordaciidae). Zool J Linn Soc 92:383–408. doi: 10.1111/j.1096-3642.1988.tb01730.x

Pardo R, Scott S, Vila I (2005) Analisis de formas en especies Chilenas del generoTrichomycterus (Osteichthyes: Siluriformes) utilizando morfometria geometrica. Gayana (Concepción) 69:180–183. doi: 10.4067/S0717-65382005000100023

Ruzzante DE, Walde SJ, Cussac VE, et al (2006) Phylogeography of the Percichthyidae (Pisces) in Patagonia: roles of orogeny, glaciation, and volcanism. Mol Ecol 15:2949–68. doi: 10.1111/j.1365-294X.2006.03010.x

Ruzzante DE, Walde SJ, Gosse JC, et al (2008) Climate control on ancestral population dynamics: insight from Patagonian fish phylogeography. Mol Ecol 17:2234–44. doi: 10.1111/j.1365-294X.2008.03738.x

Salas D, Veliz D, Scott S (2012) Diferenciación morfológica en especies del género Cheirodon ( Ostariophysi : Characidae ) mediante morfometría tradicional y geométrica. 76:142–152.

Tsuzuki MY, Strüssmann CA, Takashima F (2008) Effect of salinity on the oxygen consumption of larvae of the silversides *Odontesthes hatcheria* and *O. bonariensis* (Osteichthyes, Atherinopsidae). Brazilian Arch Biol Technol 51:563–567. doi: 10.1590/S1516-89132008000300017

Unmack PJ, Barriga JP, Battini M a., et al (2012) Phylogeography of the catfish *Hatcheria macraei* reveals a negligible role of drainage divides in structuring populations. Mol Ecol 21:942–959. doi: 10.1111/j.1365-294X.2011.05408.x

Unmack PJ, Bennin AP, Habit EM, et al (2009) Impact of ocean barriers, topography, and glaciation on the phylogeography of the catfish *Trichomycterus areolatus* (Teleostei: Trichomycteridae) in Chile. Biol J Linn Soc 97:876–892. doi: 10.1111/j.1095-8312.2009.01224.x

Vanhaecke D, Garcia de Leaniz C, Gajardo G, et al (2012) DNA barcoding and microsatellites help species delimitation and hybrid identification in endangered galaxiid fishes. PLoS One 7:e32939. doi: 10.1371/journal.pone.0032939

Vargas P V., Arismendi I, Gomez-uchida D (2015) Evaluating taxonomic homogenization of freshwater fish assemblages in Chile. Rev Chil Hist Nat. doi: 10.1186/s40693-015-0046-2

Vila I (2006) A New Species of Killifish in the Genus Orestias (Teleostei: Cyprinodontidae) from the Southern High Andes, Chile. Copeia 2006:472–477. doi: 10.1643/0045-8511(2006)2006[472:ANSOKI]2.0.CO;2

Vila I, Mendez MA, Scott S, et al (2007) Threatened fishes of the world: *Orestias ascotanensis* Parenti, 1984 (Cyprinodontidae). Environ Biol Fishes 80:491–492. doi: 10.1007/s10641-006-9150-0

Vila I, Scott S, Mendez MA, et al (2011) *Orestias gloriae*, a new species of cyprinodontid fish from saltpan spring of the southern high Andes (Teleostei: Cyprinodontidae). Ichthyol Explor Freshwaters 22:345–353.
